# From Fear to Vaccination: Changing Needs of Congenital Heart Defect Patients and Relatives over the Course of the COVID-19 Pandemic

**DOI:** 10.3390/jcm14197005

**Published:** 2025-10-03

**Authors:** Paul C. Helm, Saskia Olivia Nasri, Emily Schütte, Anna-Lena Ehmann, Janina Semmler, Felix Berger, Katharina Schmitt, Cornelia Tremblay, Julia Remmele, Stefan Orwat, Gerhard-Paul Diller, Constanze Pfitzer

**Affiliations:** 1 National Register for Congenital Heart Defects, Augustenburger Platz 1, 13353 Berlin, Germany; 2 Deutsches Herzzentrum der Charité, Department of Congenital Heart Disease—Pediatric Cardiology, Augustenburger Platz 1, 13353 Berlin, Germany; 3 Charité—Universitätsmedizin Berlin, Corporate Member of Freie Universität Berlin and Humboldt-Universität zu Berlin, 10117 Berlin, Germany; 4 Department of Obstetrics, Charité—Universitätsmedizin Berlin, Corporate Member of Freie Universität Berlin and Humboldt-Universität zu Berlin, 13353 Berlin, Germany; 5 Competence Network for Congenital Heart Defects, 13353 Berlin, Germany; 6 Deutsches Herzzentrum der Charité, Department of Psycho-Cardiology, 13353 Berlin, Germany; 7 Department of Congenital Heart Disease and Pediatric Cardiology, German Heart Center of Munich, 80636 Munich, Germany; 8 Institute of Preventive Pediatrics, Technical University Munich, 80992 Munich, Germany; 9 Adult Congenital and Valvular Heart Disease Center, Department of Cardiology and Angiology, University Hospital Muenster, 48149 Muenster, Germany

**Keywords:** congenital heart defects, COVID-19, healthcare utilization, everyday life, risk perception

## Abstract

**Background/Objectives:** As survival improves in congenital heart defects (CHD), psychosocial support—particularly during crises—has become increasingly important. We examined how concerns of CHD patients and their relatives evolved during the Coronavirus Disease 2019 (COVID-19) pandemic, focusing on the influence of role (patient vs. relative), gender, and CHD complexity. **Methods:** The German National Register for Congenital Heart Defects (NRCHD) conducted two nationwide online surveys in April 2020 (Survey 1) and April 2021 (Survey 2). Free-text responses were analyzed using Mayring’s summarizing content analysis. Categories were coded per respondent (present/absent) for exploratory comparisons by year, role, sex, and CHD complexity. Analyses were cross-sectional and descriptive (*p*-values unadjusted). **Results:** In survey 1, 15.9%, and in survey 2, 19.3% of respondents provided qualitative information. In 2020, dominant themes included general COVID-19 information (37.3%), lack of CHD-specific information (30.4%), worry (24.1%), fear (23.2%), isolation (21.4%), and uncertainty (21.2%). By 2021, concerns shifted toward vaccination (24.1%) and vaccination prioritization (23.4%), while information gaps (21.8%) and fear (21.0%) persisted. Significant year-to-year changes included decreases in general information needs, concern, isolation, and uncertainty, and increases in prioritization (all *p* < 0.01). Relatives consistently reported higher psychological burden than patients (*p* ≤ 0.01). **Conclusions:** Concerns moved from early fear/uncertainty to vaccination and prioritization one year later, with persistent information needs across subgroups. Clear CHD-specific communication, caregiver-inclusive psychosocial support, and crisis-resilient care pathways (including telemedicine) are essential for this vulnerable population.

## 1. Introduction

Congenital heart defects (CHD) are the most common congenital anomalies, affecting approximately 1% of live births globally [1]. Individuals with CHD often require lifelong medical care, with varying degrees of clinical complexity, and are thus especially vulnerable to systemic disruptions such as those caused by a global pandemic. During the COVID-19 pandemic, chronically ill individuals faced not only higher medical risks but also significant psychosocial burdens due to social isolation, disrupted healthcare access, and the proliferation of uncertain or conflicting information [2,3].

The World Health Organization (WHO) emphasized the critical importance of clear, timely communication during the pandemic, particularly for high-risk populations [4]. The WHO advises regularly updating action plans to reflect evolving situations and societal needs. Effective, comprehensive responses to crises like COVID-19 require close coordination with authorities, partners, and community networks. Proactive communication and continual assessment of vulnerable populations are essential to share accurate information, reduce stigma, and adapt strategies as conditions change [4]. This perspective was underscored by infodemic management concepts, which recommend evidence-based risk communication and community engagement for vulnerable groups [5]. Studies in other chronically ill populations have demonstrated that inadequate communication and lack of tailored health guidance exacerbated anxiety, reduced treatment adherence, and undermined trust in healthcare systems. Among adults with congenital heart disease (ACHD), anxiety is common and often co-occurs with depression. Dual elevations are linked to reduced work/study participation and lower quality of life, while many affected patients are not receiving mental healthcare [6]. Fekadu et al. found that the COVID-19 pandemic significantly disrupted the diagnosis, treatment, and follow-up care for patients with chronic diseases, especially in resource-limited settings, highlighting the impact on healthcare services and emphasizing the need for improved chronic disease management and policy interventions to reduce the burden in future crises [7].

For CHD patients and their families, these challenges are intensified by the already complex nature of their medical needs and the emotional demands of managing a chronic, potentially life-threatening condition. Even before the COVID-19 pandemic, care for CHD patients in Germany was suboptimal, particularly for ACHD. Data from 2017 to 2021 highlighted the key role of GPs in ACHD care by showing that nearly half of 4493 ACHD respondents were unaware of certified specialists or centers [8]. Diller et al. also found higher mortality risks, especially in moderate to complex CHD cases, when patients lacked cardiac follow-up through primary care [9]. These structural shortcomings existed pre-pandemic and were further exacerbated during the COVID-19 crisis.

Since March 2020, the COVID-19 pandemic has led to a decline in early and follow-up care and specialist visits due to fear of infection, with decreases of up to 30% (cardiology) and 50% (oncology) [10]. A study of 2047 chronically ill patients (April 2020 to November 2021) found that over 20% skipped at least one preventive service, with over 40% of cancelations due to fear of COVID-19 [11]. In 2020, data from the German Central Institute for the Association of Statutory Health Insurance Physicians and AOK (a network of 11 independent health insurance funds) revealed a marked decline in outpatient and inpatient treatments, particularly during the first lockdown: 39% fewer inpatients overall, a 31% decrease for acute myocardial infarction, and a 50% decrease for chronic ischemic heart disease [12,13]. CHD-specific analyses are lacking, as ICD-10 codes do not adequately capture the condition [14].

Understanding the lived experiences of CHD patients and their relatives during different stages of the pandemic is essential for improving preparedness and patient-centered care strategies in future crises. This study aims to analyze qualitative responses from two large-scale online surveys conducted at the beginning of the pandemic (April 2020) and one year later (April 2021) in Germany.

## 2. Materials and Methods

### 2.1. Study Design and Patient Cohort

The analyses are based on two online surveys conducted by the National Register for Congenital Heart Defects (NRCHD) on COVID-19, involving registered patients or their relatives. Both were exploratory cross-sectional studies with quantitative and qualitative analyses. The importance of the NRCHD for health services research and transition-of-care topics has been repeatedly described [15,16,17,18,19,20,21].

Survey 1, conducted in April 2020 (anonymous, 49 questions, ~20 min), addressed healthcare provision, information level, infection risk, and disease-specific risk assessment, with 3558 participants, 15.9% of whom provided free-text comments. Survey 2, conducted in April 2021 (pseudonymized, 78 questions, ~30 min), added topics such as testing, vaccination, preventive measures, and past infections, with 3179 participants, 19.3% of whom gave free-text input. Overlap between participant groups is possible but cannot be identified.

Eligible invitees were NRCHD members with a documented congenital heart defect (patients) and their relatives who had consented to be contacted for research. Participation was voluntary and online. No restrictions on age, sex, or CHD severity were applied. Additional requirements were a valid email/postal address, German language proficiency, and internet access. No incentives were provided.

### 2.2. National Register for Congenital Heart Defects

Founded in 2003 by German cardiology societies, the NRCHD collects nationwide data on patients with CHD, documenting prevalence, disease progression, care, and quality of life, and serving as a basis for research projects. As of March 2025, around 60,000 patients or relatives were registered. Participation is voluntary. The first survey (April 2020) was conducted anonymously under the general ethics approval (EA2/040/23). The second survey (April 2021) was pseudonymized and received separate ethics approval (EA2/109/21).

### 2.3. Statistical Analyses

Empirical social research distinguishes between quantitative methods for statistical analysis of measurable phenomena and qualitative methods for in-depth study of social experiences [22,23]. This study employed a mixed-methods approach to combine the strengths of both and validate results [22,24,25,26]. Qualitative data from open-ended questions were transformed into quantitative form (“quantification”) to enable comparability [27,28]. Data were primarily collected via open-ended survey questions [23,29]. The qualitative analysis followed an exploratory approach based on Mayring to capture participants’ perspectives and lived experiences during the COVID-19 pandemic [30,31].

Mayring’s qualitative content analysis relies on a category system developed deductively beforehand or inductively during analysis [30,31]. This structured approach ensures transparency, intersubjectivity, and comparability [30]. In this study, the summarizing content analysis was applied, condensing large volumes of material to derive core statements and an initial category system [29,32]. The system is progressively generalized, repeatedly checked against the data, and, if necessary, refined in a cyclical process [30] referring to internationally established approaches [33] and to the concept of quantizing qualitative data [28].

The qualitative survey data were exported from SPSS (Version 29) to Excel and organized in tables. Clear, topic-related text elements served as coding units. Surveys 1 and 2 were analyzed separately, starting with Survey 1. After repeated readings, thematic clusters were identified. These were repeatedly reviewed, expanded, and condensed into main categories. Following Mayring’s summarizing content analysis [30], two analysts independently conducted iterative coding cycles (paraphrase, reduction, abstraction) to develop and refine a shared category system. We applied double coding with negotiated agreement: both coders coded the same batches, then met to reconcile discrepancies, refine code definitions, and update the codebook. A senior researcher performed periodic spot checks and resolved rare escalations. Because our goal was iterative category development in an exploratory framework, we did not compute a coefficient (e.g., Cohen’s κ). Instead, we prioritized intersubjective traceability and consensus through analyst triangulation, decision logs, and exemplar anchoring. We acknowledge that the absence of a coefficient is a limitation; however, negotiated agreement is appropriate in summarizing content analysis focused on transparent category construction rather than hypothesis testing.

Finally, a frequency analysis was conducted to weight the relevance of individual perspectives; categories with the highest assignment rates were used for quantification [34]. After coding, the qualitative data were converted into quantitative values by assigning and counting core statements within the main categories. Each mention of a category was coded as 1, and each non-mention as 0.

Quantitative data from the transformed qualitative statements were analyzed using IBM SPSS 29. Results are reported in absolute and relative frequencies, percentages, and, where applicable, means and standard deviations, presented in text, tables, and charts. Unless noted otherwise, percentages refer to demographic/medical patient data or to the proportion of free-text respondents in each survey year who mentioned a category at least once (present vs. absent). Group comparisons use the respective subgroup denominators. Group comparisons were made by respondent type, sex, CHD severity (Warnes et al. [35]), and between survey periods. Depending on the scale level, chi-square, Mann–Whitney U, or *t*-tests were applied (significance level *p* ≤ 0.05). As exploratory cross-sectional studies, *p*-values are considered exploratory; no adjustment for multiple testing was performed to avoid missing potential differences.

## 3. Results

### 3.1. Study Cohort/Patient Characteristics

10,443 people were invited to Survey 1 and 27,145 to Survey 2. A total of 6737 CHD patients/relatives were analyzed (overall response rate 17.9%. In both surveys, more patients than relatives completed the questionnaire (see Table 1).

Survey 2 patients were older than those in Survey 1 (*p* < 0.001) and most lived in former West German states with significant differences emerged in settlement size (*p* < 0.001) and education (see Table 1).

Gender differences appeared in both surveys: women more often completed the survey themselves (*p* < 0.001), were older, more often part-time employed. Men were younger, more often still in school, and more frequently in full-time employment. A detailed overview can be found in Table 1.

### 3.2. Medical and Disease-Related Patient Data

For over 91% of patients, CHD severity could be classified. Between 2020 (Survey 1) and 2021 (Survey 2), simple CHD decreased while moderate and complex CHD increased (see Table 2).

Since the pandemic began, medical visits have increased markedly, especially to cardiologists in practices and hospitals (*p* < 0.001). Hospitalizations for CHD were more frequent in 2021. Self-canceled appointments “due to COVID-19” were around 8 to 10%, cancelations by doctors remained stable. In 2021, more patients discussed COVID-19 with doctors (*p* < 0.001), more often without prompting or upon request. Use of medical hotlines rose sharply as COVID-19 cases in close contacts and self-reported infections did (see Table 2).

Influenza vaccination rates increased (*p* < 0.001), pneumococcal vaccination remained stable (around 54%). COVID-19 was more often the reason for vaccination in 2021. Comorbidities such as chronic lung disease or diabetes were rare (around 8 to 9%), as were immunodeficiency (around 5%) and immunosuppressive therapy (around 5%). A detailed overview can be found in Table 2.

### 3.3. Qualitative Answers, Quantitatively Categorized

In the qualitative free-text section, 15.9% of participants in Survey 1 (*n* = 565) and 19.3% in Survey 2 (*n* = 615) contributed responses. Statements were grouped into 16 categories via paraphrase/reduction cycles and aligned with survey prompt. In Survey 2, “Vaccination” and “COVID-19 infection” were added to capture emergent 2021 topics, while “Consequences” remained specific to 2020 responses.

Highly significant differences (*p* < 0.001) were found in five categories: general COVID-19 information level (37.3% vs. 26.2%), concerns (24.1% vs. 12.2%), isolation (21.4% vs. 13.7%), uncertainty (21.2% vs. 8.5%), and prioritization (15.2% vs. 23.4%). A moderate difference (*p* < 0.01) appeared for insufficient information (30.4% vs. 21.8%).

“COVID-19 vaccination” (24.1%) and “COVID-19 infection” (5%) were mentioned only in 2021, while “Consequences” appeared only in 2020 (3.4%).

Only two slight significant gender differences appeared in the main categories: In Survey 1, the “positive & unconcerned” category was more frequent among females (16.5% vs. 10.2%; *p* < 0.05) and in Survey 2, female patients or their relatives expressed fear/anxiety more often than males (24.6% vs. 17.1%; *p* < 0.05). All other categories showed no significant gender differences.

#### 3.3.1. Survey 1 (April 2020)

In Survey 1, the 565 study participants most frequently (37.3%/30.4%) reported information about COVID-19 in general and insufficient information about COVID-19 in the context of CHD, with worries (24.1%) being the third most frequently reported, closely followed by explicitly expressed fears related to COVID-19 (23.2%). Details are shown in Table 3.

Patients and relatives differed significantly in 5 of the 14 topics identified in Survey 1. All five topics were addressed significantly more frequently by the relatives than by the patients themselves (*p* < 0.001 to *p* < 0.05; see Table 3).

In comparison, the opportunity to express opinions was used primarily when a moderate or complex CHD was present [no qualitative responses: simple CHD (27%), moderate CHD (36.6%), complex CHD (29.1%), other CHD (7.3%); qualitative responses: simple CHD (19.6%), moderate CHD (34.9%), complex CHD (37.9%)]. In seven topics, there were significant differences in the frequency of the topics mentioned between the CHD severity levels (*p* < 0.001 to *p* < 0.05; see Table 3).

#### 3.3.2. Survey 2 (April 2021)

In Survey 2, the 615 study participants most frequently (26.7%) reported information about COVID-19 in general, with vaccination (24.1%) being the second most frequently reported, closely followed by prioritization (23.4%) and insufficient information status about COVID-19 in context with the CHD. Details are shown in Table 4.

In Survey 2, patients and relatives differed significantly in 5 of the 15 topics identified and four of the five topics were addressed significantly more frequently by the relatives than by the patients themselves (*p* < 0.01 to *p* < 0.05; see Table 4).

In Survey 2, in comparison, the opportunity to express opinions was used primarily when a moderate or complex CHD was present [no qualitative responses: simple CHD (14.4%), moderate CHD (40.1%), complex CHD (36.5%), other CHD (9.1%); qualitative responses: simple CHD (14.6%), moderate CHD (35.3%), complex CHD (43.1%)]. In five topics, there were significant differences in the frequency of the topics mentioned between the CHD severity levels (*p* < 0.001 to *p* < 0.05; see Table 4).

## 4. Discussion

Two nationwide online surveys (April 2020 & 2021, *n* = 6737) of CHD patients and relatives collected quantitative and qualitative data on the impact of the COVID-19 pandemic. The quantitative results of this study have been published previously [19]. Full descriptions of the sample and disease-related clinical data are available there. Accordingly, only a brief overview is provided here, and the analysis centers on qualitative perspectives from CHD patients and their relatives.

In the qualitative responses (Survey 1: *n* = 565; Survey 2: *n* = 615), general COVID-19 information was the most cited topic in both years (37.3%/26.2%). In 2020, insufficient information (30.4%), worries (24.1%), fear/anxiety (23.2%), and isolation (21.4%) followed. In 2021, key topics shifted to vaccination (24.1%), prioritization (23.4%), insufficient information (21.6%), and fear/anxiety (21%). Initially, information deficits, worries, and fears dominated.

Relatives consistently reported higher rates of fear, isolation, and psychological burden than patients in both years. This likely reflects caregiving strain, role overload during school/child-care closures, and heightened anticipatory anxiety when advocating for medically vulnerable family members. Our subgroup patterns (higher fear/isolation among relatives; sustained psychological burden) underscore the need to target communication and psychosocial supports to families/caregivers, not only patients.

Similar patterns were observed in cohorts of ACHD, in which burden measures increased significantly during the pandemic [36]. After one year, the focus shifted to protective measures and perceived neglect of vulnerable groups. CHD severity, biological sex, and perspective (patient vs. relative) each significantly influenced responses. The pandemic significantly negatively affected medical care for CHD patients.

The trajectory—from early fear/uncertainty and information deficits to later vaccination/prioritization—aligns with reports from other settings. In the United Kingdom, parents of children with CHD during the first wave described marked gaps in official support and CHD-specific information, alongside difficulties navigating care and guidance under lockdown [37]. Across adult congenital cohorts, psychological distress remained elevated as the pandemic progressed, with notable age/sex differences, reinforcing the persistence of psychosocial need even as clinical priorities shifted [36]. In our 2021 wave, concerns pivoted toward vaccination access and prioritization against a backdrop of ongoing information needs; taken together, these strands suggest that the observed transition from fear/uncertainty to vaccination/prioritization likely extends beyond Germany, while the concrete prioritization pathways remain system-specific [38].

### 4.1. Study Cohort/Patient Characteristics

Respondents came from across Germany, mostly from the former West German states. In both surveys, nearly 70% of participants were from the old federal states (former West Germany). Consequently, participants from the new federal states (former GDR) are slightly overrepresented relative to the national population, where roughly 80% of residents live in the old federal states [39]. In Survey 1, participants more often lived in small/medium towns (≤5000 inhabitants: 31.1% vs. 22.1% in Survey 2). Both surveys are large registry-based samples (*n* = 6737; Survey 1: 3558; Survey 2: 3179). The response rate dropped from 34.1% to 11.7%, likely due to habituation and pandemic-related “COVID fatigue”. Female patients/relatives were slightly overrepresented (53%/52.6%), consistent with studies showing women’s greater online survey participation [40].

In both surveys, more patients than relatives completed the questionnaire (57%/66.2%; *p* < 0.001), likely reflecting the large proportion of ACHD in Germany [41] and an increased need to share experiences during the pandemic [42]. Lower perceived COVID-19 risk for children/adolescents (e.g., mortality 0.08% in 7480 infected children, [43]) may explain reduced relative participation—particularly for simple CHD cases. Female patients more often completed the survey themselves than males (Survey 1: 64.4% vs. 48.7%; Survey 2: 71.3% vs. 60.7%). The gender gap in self-response decreased from 15.7% to 10.6%, amid an overall rise in self-participation.

### 4.2. Medical and Disease-Related Patient Data

CHDs were classified by Warnes et al. [35] into simple, moderate, and complex CHD. In both surveys, fewer simple CHD cases participated (2020: 25.8%; 2021: 14.2%), while moderate (36.3% vs. 39.1%) and complex CHD (30.5% vs. 37.7%) remained stable or increased. The drop in simple CHD may reflect lower health risk and reduced participation interest after one pandemic year.

Complex CHD patients were already accustomed to infection control measures pre-pandemic [44,45,46] and often had established medical contacts. Their largest participation increase may relate to rising infection rates and limits of prolonged self-isolation.

Simple CHD was more frequent in women (2020: 30.6% vs. 20.4%; 2021: 17.5% vs. 10.6%), possibly due to greater distress, stronger interest in COVID-19, or heightened health awareness [47]. Moderate CHD showed balanced gender ratios and complex CHD were more common in male patients.

By 2021, more participants had seen a physician. Self-cancelations “due to COVID-19” rose slightly (8% vs. 9.6%; *p* < 0.01); surgery/catheter cancelations remained <1%. Doctor-initiated cancelations stayed at ~10%. Similar care disruptions are reported in oncology [48,49]. Akkermann et al. [50] likewise report effects on medical care and appointment avoidance among ACHD during the COVID-19 pandemic.

### 4.3. Qualitative Answers, Quantitatively Categorized

In the qualitative free-text section, 565 participants (15.9%) responded at the pandemic’s start and 615 (19.3%) after one year; the share of relatives fell from 39.1% to 30.9%. Women were more frequent respondents. Analysis following Mayring et al. [30] identified 16 main categories, 13 comparable across surveys. The most frequent theme was general COVID-19 information (37.3% vs. 26.2%; *p* < 0.001), especially insufficient information (30.4% vs. 21.8%; *p* < 0.01). “Good information” was rare (7.5%/5%). These findings align with Döpfmer et al. [51] on inadequate communication.

Early in the pandemic, concern (24.1% vs. 12.2%; *p* < 0.001), fear (23.2% vs. 21%), isolation (21.4% vs. 13.7%; *p* < 0.001), and uncertainty (21.2% vs. 8.5%; *p* < 0.001) dominated. Declines likely reflect positive experiences over time, though systemic weaknesses persisted [52].

Vaccination emerged only in 2021 (24.1%) and was closely linked to prioritization (15.2% vs. 23.4%), as CHD patients were often not explicitly considered in official plans [53,54]. Psychological burden remained steady (around 14.5%). “Consequences” (3.4%) appeared only in 2020; “COVID-19 infection” (5%) only in 2021.

#### 4.3.1. Patients and Relatives

In both surveys, more patients than relatives contributed to the free-text section (2020: 344 vs. 221; 2021: 425 vs. 190). Relatives were proportionally more represented in most main categories, notably “fear/anxiety” (2020: 33% vs. 16.9%; 2021: 25.8% vs. 18.8%). Higher fear among relatives reflects parental concern for children with CHD, personal health risks, and isolation burdens. Reassuring studies on COVID-19 in children [55,56] may reduced concern, especially for mild CHD cases.

Relatives cited isolation about twice as often as patients (2020: 30.3% vs. 15.7%; 2021: 21.1% vs. 10.4%; *p* < 0.001), partly because chronic patients often experienced isolation pre-pandemic [57], while relatives faced it for the first time.

Concern remained higher among relatives (2020: 28.5% vs. 21.2%; 2021: 18.4% vs. 9.4%). Psychological burden was also more common in relatives (2020: 19.5% vs. 11.3%; 2021: 22.6% vs. 10.6%), indicating the pandemic amplified existing family challenges. Conversely, in 2021 patients were more represented in the “positive/unconcerned” category, consistent with other findings.

#### 4.3.2. CHD Complexity

At the pandemic’s start, significant differences between CHD severity groups appeared in seven main categories (incl. fear, concern, uncertainty, isolation, psychological burden, prioritization); after one year, in four (fear, psychological burden, prioritization, vaccination). “Positive/unconcerned” was more frequent in simple CHD, while mentions of other categories increased with CHD severity—consistent with greater physical, psychosocial, and occupational burdens in complex CHD [58,59].

Care gaps for ACHD patients existed pre-pandemic [60,61,62]. In 2020, fear was three times higher in complex than simple CHD (31.8% vs. 11.7%; *p* < 0.001), with moderate CHD in between (21.3%). By 2021, fear in complex CHD fell markedly (24.2%), possibly due to vaccination and better information. Psychological burden was twice as high in complex vs. moderate and four times higher than in simple CHD (22% vs. 11.2%/6.3%; *p* < 0.001) and remained higher in 2021.

Concern was over twice as high in complex vs. simple CHD (32.7% vs. 14.4%; *p* < 0.01). Prioritization issues were more frequent in complex CHD but rose across all groups, most in simple CHD (7.2% vs. 16.9%), likely due to STIKO-based vaccination schedules [63] that did not explicitly include CHD, creating access barriers to higher priority tiers.

Recent data from Diller et al. [64] indicate increased infection susceptibility in one-third of CHD patients and immune defects in 5.6% (vs. 2.9% in healthy controls), suggesting a relevant risk of severe infection regardless of CHD complexity.

#### 4.3.3. Gender Aspects

Female participants slightly outnumbered males in both surveys (2020: 321 vs. 244; 2021: 317 vs. 298). A significant gender difference appeared only in 2021 for “fear” (female 24.6% vs. male 17.1%; *p* < 0.05). In 2020, the female share was also higher (24% vs. 22.1%) but not significant. Notably, women, despite higher fear levels, also more often reported being “positive & unconcerned.” No other categories showed significant gender differences, suggesting gender played a minor role in shaping responses.

### 4.4. Lessons Learned?

Based on this study’s findings, the WHO’s call for regularly updated action plans, coordinated partnerships, proactive communication, and ongoing assessments [4] remains highly relevant but is often overlooked or ignored in practice.

The qualitative trajectory observed here directly informs practice. Information gaps in 2020 argue for pre-approved, CHD-tailored RCCE assets deliverable via registries/centers at crisis onset. Persistent fear/uncertainty suggests proactive counseling scripts for clinicians (risk framing, access routes, coping resources). The 2021 focus on vaccination/prioritization highlights the policy interface: specialty societies and registries should maintain ready-to-submit briefs to ensure chronic cardiac conditions are visibly accommodated in prioritization schemes. Finally, sustained psychological burden among relatives supports embedding structured psychosocial triage and referral in both in-person and telemedicine encounters.

The growing ACHD population and COVID-19-driven service shifts underscore the need for patient-centered, scalable care. We recommend an evidence-based telemedicine framework for ACHD, with implementation guidance to ensure effective, equitable, and continuous specialist care—even during crises such as a global pandemic. Early-pandemic experience was encouraging: in Dodeja et al. [65], 98% of ACHD patients felt their needs were met, and only 15% reported technical issues, suggesting telemedicine can serve as a viable, patient-preferred alternative to in-person visits when appropriate. Borrelli et al. [66] also note that ACHD now outnumber children with CHD and that COVID-19 accelerated telemedicine’s role as a core care model; they advocate an integrated, patient-centered digital-care strategy tailored to ACHD patients’ unique needs [66].

Nonetheless, some scenarios require in-person specialist examination or inpatient/surgical care. Age, communication ability, and clinical complexity also influence whether patients can adequately articulate concerns via video. Telemedicine should therefore be one component of a broader, holistic care model. Further studies are needed to define approaches that guarantee comprehensive, high-quality ACHD care in everyday practice and during crises.

### 4.5. Policy Linkage

Our findings reveal a clear qualitative trajectory from early fear and uncertainty and CHD-specific concerns to later concerns about vaccination access and prioritization. This evolution highlights three actionable policy levers:

♦ Communication: Develop and pre-approve CHD-tailored RCCE materials (e.g., FAQs, risk framing, vaccination guidance) for rapid deployment (within 48 h) by registries and specialist centers, with updates aligned to national advisories.
♦ Prioritization: Create formal pathways to include chronic cardiac conditions (e.g., complex CHD, immunodeficiency) in vaccination and therapy prioritization schemes, using clinician attestation templates to streamline access.
♦ Access Continuity: Implement structured telemedicine triage systems and registry-enabled alerts (e.g., hotline availability, protected appointment slots) to ensure uninterrupted care.

### 4.6. Clinical Implications

To translate our findings into actionable practice, we recommend the following steps for healthcare providers and systems:

1. Implement a concise, standardized counseling script for all CHD patient interactions, covering:
  ♦ Risk communication tailored to CHD complexity
  ♦ Vaccination recommendations and prioritization verification
  ♦ Clear return-to-care plans
2. Flag patients with complex CHD or immunodeficiency for:
  ♦ Proactive outreach (e.g., phone/email check-ins)
  ♦ Expedited in-person reviews during crises
3. Psychosocial Screening and Support:
  ♦ Routinely screen both patients and relatives for psychological burden
  ♦ Provide warm handoffs to psychosocial services for those in need
4. Adopt a telemedicine-first approach, with predefined escalation triggers for urgent in-person care, such as:
  ♦ New or worsening cyanosis
  ♦ Signs of heart failure
  ♦ Pregnancy-related concerns
  ♦ Peri-procedural questions
  ♦ Ensure a protected same-week clinic slot for escalated cases
5. Deferred Care Tracking
  ♦ Document all deferred care and maintain recall lists to ensure follow-up

Embedding these steps into routine workflows will operationalize our findings, fostering crisis-resilient, family-inclusive CHD care during future healthcare emergencies.

### 4.7. Limitations

Findings are only partly transferable internationally, as Germany’s healthcare system and country-specific pandemic responses differ widely. Parallels exist mainly with certain EU countries, but comparability remains limited.

Uneven participation rates between patients and relatives limit such subgroup comparisons. However, high sample sizes (Survey 1: 3558; Survey 2: 3179; qualitative: 565 and 615) are sufficient for a reliable snapshot.

Response rates declined from 34.1% to 11.7% between surveys, introducing potential selection bias toward more engaged or digitally connected participants and possibly under-capturing less-concerned individuals in 2021. Findings should therefore be interpreted as snapshots of engaged NRCHD members rather than population prevalence estimates. Possible explanations for the declined response rates could be acclimatization with the pandemic and pandemic fatigue on the one hand and a more extensive list of questions in survey 2 on the other. However, the sample sizes for both survey 1 and survey 2 are sufficiently high to provide robust results. All statistical tests are exploratory and no correction for multiple comparisons was performed to avoid missing potentially relevant differences.

Since Survey 1 and 2 participants need not be the same, results are cross-sectional, not longitudinal. No rural–urban distinction was made, although pre-pandemic access to specialist care was already more limited in rural areas [67].

This study relied solely on two online surveys, which may have inadvertently excluded specific groups, such as older individuals or those with limited internet access. The decision to use an online format was driven by the constraints imposed during the pandemic, enabling efficient data collection across a wide geographical area. While this approach facilitated the inclusion of a diverse and extensive sample of congenital heart disease (CHD) patients and their families, it may not fully represent the perspectives of marginalized or disadvantaged groups who faced participation barriers. Consequently, the findings could be subject to selection bias, potentially limiting their applicability to the entire CHD population. Despite these limitations, the large sample size enhances the credibility and robustness of the results. It is important to note that, as cross-sectional studies, the findings only establish correlations and do not infer causality or directional effects.

## 5. Conclusions

This two-phase nationwide analysis underscores the dynamic informational and emotional needs of CHD patients and their families during the COVID-19 pandemic. While initial responses focused on uncertainty and fear, concerns later shifted toward systemic responses such as vaccination and prioritization. Significant differences between patient and caregiver perspectives, and across CHD severity levels, point to the need for targeted, inclusive health communication. The findings advocate for more resilient, patient-centered healthcare systems equipped to support vulnerable groups not only during pandemics but in all future crises.

## Figures and Tables

**Table 1:** Study Cohort/Patient Characteristics.

Characteristic	Survey 1 (2020)	Survey 2 (2021)	Notable Differences/*p*-Value
Invited/Participants	10,443 invited 3558 participants	27,145 invited 3179 participants	Overall response rate 17.9% Survey 1: 34.1% Survey 2: 11.7%
Role (patients/relatives)	Patients 57%	Patients 66.2%	↑ patient share *p* < 0.001
Female patients	~53%	~53%	—
Mean patient age (years)	23.7 ± 15.4	26.0 ± 16.1	↑ age *p* < 0.001
	Women’s age 24.7 ± 14.9 vs. men 22.4 ± 16	Women’s age 26.4 ± 15.3 vs. men 25.6 ± 16.9	↑ Women vs. men in survey 1 *p* < 0.001
Residence: former West Germany	69.6%	68.7%	—
Settlement ≤5000 inhabitants	31.1%	22.1%	↓ small-town residence *p* < 0.001
Overall self-completed survey by patients	57% self completed	66.2% self-completed	↑ overall self-completed *p* < 0.001
Self-completed survey by patients gender	Women more often self-completed (64.4% vs. men 48.7%)	Women more often self-completed (71.3% vs. men 60.7%)	↑ Women vs. men between survey 1/2 and within both surveys *p* < 0.001
Education “not yet in school”	14.8%	11%	↓ (*p* < 0.001)
Employment “still in school”	Women less often still went to school (23.9% vs. men 32.6%)	Women less often still went to school (23.6% vs. men 28.6%)	↓ Women vs. men in both surveys *p* < 0.001
Employment “part-time”	Women more often employed in part-time (13.4% vs. men 2.9%)	Women more often employed in part-time (12.8% vs. men 3.5%)	↑ Women vs. men in both surveys *p* < 0.001

↑ Increase between the two surveys, ↓ Decrease between the two surveys.

**Table 2:** Medical and Disease-Related Patient Data.

Characteristic	Survey 1 (2020)	Survey 2 (2021)	Notable Differences/*p*-Value
CHD severity	Simple CHD 25.8%	Simple CHD 14.2%	↓ (*p* < 0.001)
	Moderate CHD 36.3%	Moderate CHD 39.1%	↑ (*p* < 0.001)
	Complex CHD 30.5%	Complex CHD 37.7%	↑ (*p* < 0.001)
Cardiologists/pediatric cardiologists (medical practice)	24.4%	39.8%	↑ (*p* < 0.001)
	Women less often 21.8% vs. men 27.2%	Women less often 37.2% vs. men 42.6%	Survey 1: ↓ (*p* < 0.01) Survey 2: ↓ (*p* < 0.05)
Cardiologists/pediatric cardiologists (clinic/heart center)	23.1%	46.6%	↑ (*p* < 0.001)
Hospitalizations for CHD	5.0%	14.3%	↑ (*p* < 0.001)
Self-canceled medical appointments due to COVID-19	~8–10%	~8–10%	Stable
Discussed COVID-19 with doctor	No discussion 50.4%	No discussion 39.0%	↑ More discussions (*p* < 0.001)
	Discussion initiated without prompting (7.8%)	Discussion initiated without prompting (17.8%)	↑ (*p* < 0.001)
	Discussion upon request (15.5%)	Discussion upon request (27.6%)	↑ (*p* < 0.001)
Use of medical hotlines	6.4%	21.1%	↑ (*p* < 0.001)
Influenza vaccination	50.9%	58.5%	↑ (*p* < 0.001)
Vaccination due to COVID-19	4.9%	9.7%	↑ (*p* < 0.001)
Comorbidities (lung disease/diabetes)	~8–9%	~8–9%	Stable
Immunodeficiency/Immunosuppressive therapy	~5%	~5%	Stable
COVID-19 cases in close contacts	12.3%	63.5%	↑ (*p* < 0.001)
Self-reported COVID-19 infection	0.4%	4.2%	↑ (*p* < 0.001)

↑ Increase between the two surveys, ↓ Decrease between the two surveys.

**Table 3:** Frequency of topics mentioned in relation to the COVID-19 pandemic in Survey 1 (April 2020): comparisons between patients and relatives, across CHD severity levels, and by percentage of qualitative responders (category mentioned vs. not mentioned). Percentages for categories refer to the subset who provided free-text in that year (presence/absence coding). Color coding from highly relevant (red) to less relevant (blue). CHD = congenital heart defects; COVID-19 = Coronavirus Disease 2019.

	Total (*n* = 565)	Patients (*n* = 344)	Relatives (*n* = 221)	*p*-Value	Simple CHD (*n* = 111)	Moderate CHD (*n* = 197)	Complex CHD (*n* = 214)	*p*-Value
information situation	37.3%							
information situation (insufficient)	30.4%							
Worry	24.1%	21.2%	28.5%	<0.05	14.4%	21.8%	32.7%	<0.01
Fear/Anxiety	23.2%	16.3%	33.0%	<0.001	11.7%	21.3%	31.8%	<0.001
Isolation	21.4%	15.7%	30.3%	<0.001	9.0%	20.8%	27.1%	<0.01
Uncertainty/Insecurity	21.2%				15.3%	18.8%	27.1%	<0.05
COVID-19 measures	16.1%							
Prioritization	15.2%				7.2%	15.2%	19.2%	<0.05
Psychological stress	14.5%	11.3%	19.5%	<0.01	6.2%	11.2%	22.0%	<0.001
Positive & unconcerned	13.8%				22.5%	12.2%	9.8%	<0.01
Medical care	12.2%							
Information situation (good)	7.6%							
Other notes	5.5%	2.2%	10.4%	<0.001				
Consequences	3.4%							

**Table 4:** Frequency of topics mentioned in relation to the COVID-19 pandemic in Survey 2 (April 2021): comparisons between patients and relatives, across CHD severity levels, and by percentage of qualitative responders (category mentioned vs. not mentioned). Percentages for categories refer to the subset who provided free-text in that year (presence/absence coding). Color coding from highly relevant (red) to less relevant (blue). CHD = congenital heart defects; COVID-19 = Coronavirus Disease 2019.

	Total (*n* = 615)	Patients (*n* = 425)	Relatives (*n* = 190)	*p*-Value	Simple CHD (*n* = 89)	Moderate CHD (*n* = 217)	Complex CHD (*n* = 265)	*p*-Value
information situation	26.7%							
Vaccination	24.1%				19.1%	20.3%	29.8%	<0.05
Prioritization	23.4%				16.9%	18.9%	29.4%	<0.01
Information situation (insufficient)	21.8%							
Fear/Anxiety	21.0%				11.2%	18.9%	24.2%	<0.05
COVID-19 measures	17.7%							
Psychological stress	14.3%	10.6%	22.6%	<0.001	3.4%	11.5%	19.6%	<0.001
Isolation	13.7%	10.4%	21.1%	<0.001				
Positive & unconcerned	13.7%	16.5%	7.4%	<0.01	30.3%	13.4%	8.3%	<0.001
Worry	12.2%	9.4%	18.4%	<0.01				
Medical care	10.1%							
Uncertainty/Insecurity	8.5%							
Information situation (good)	5.0%							
COVID-19 infection	5.0%							
Other notes	4.9%	1.9%	11.6%	<0.001				

## Data Availability

Data cannot be shared for data protection reasons.
